# Relationship of the lung microbiome with PD-L1 expression and immunotherapy response in lung cancer

**DOI:** 10.1186/s12931-021-01919-1

**Published:** 2021-12-28

**Authors:** Hye Jin Jang, Ji Yeon Choi, Kangjoon Kim, Seung Hyun Yong, Yeon Wook Kim, Song Yee Kim, Eun Young Kim, Ji Ye Jung, Young Ae Kang, Moo Suk Park, Young Sam Kim, Young-Jae Cho, Sang Hoon Lee

**Affiliations:** 1 Division of Pulmonology, Department of Internal Medicine, Institute of Chest Diseases, Severance Hospital, Yonsei University College of Medicine, 50-1 Yonsei-ro, Seodaemun-gu, Seoul, 03722 Republic of Korea; 2 Division of Pulmonary and Critical Care Medicine, Department of Internal Medicine, Seoul National University Bundang Hospital, 82 Gumi-ro, 173 Beon-gil, Bundang-gu, Seongnam-si, Gyeonggi-do 463-707 Republic of Korea

**Keywords:** Human microbiome, Taxonomy, Lung cancer, PD-L1 expression levels

## Abstract

**Background:**

Lung cancer is the primary cause of cancer-related deaths worldwide. The human lung serves as a niche to a unique and dynamic bacterial community that is related to the development of multiple diseases. Here, we investigated the differences in the lung microbiomes of patients with lung cancer.

**Methods:**

16S rRNA sequencing was performed to evaluate the respiratory tract microbiome present in the bronchoalveolar lavage fluid. Patients were stratified based on programmed death-ligand 1 (PD-L1) expression levels and immunotherapy responses.

**Results:**

In total, 84 patients were prospectively analyzed, of which 59 showed low (< 10%), and 25 showed high (≥ 10%) PD-L1 expression levels. The alpha and beta diversities did not significantly differ between the two groups. *Veillonella dispar* was dominant in the high-PD-L1 group; the population of *Neisseria* was significantly higher in the low-PD-L1 group than in the high-PD-L1 group. In the immunotherapy responder group, *V. dispar* was dominant, while *Haemophilus influenzae* and *Neisseria perflava* were dominant in the non-responder group.

**Conclusion:**

The abundances of *Neisseria* and *V. dispar* differed significantly in relation to PD-L1 expression levels and immunotherapy responses.

**Supplementary Information:**

The online version contains supplementary material available at 10.1186/s12931-021-01919-1.

## Background

Lung cancer is the main cause of cancer-related deaths worldwide. In 2020, 228,820 cases reported in the United States were attributed to lung cancer, which was approximately a quarter of all reported cancer-related deaths [1, 2]. Targeted therapy for biomarkers, such as epidermal growth factor receptor, anaplastic lymphoma kinase, receptor tyrosine kinase ROS1, receptor tyrosine kinase RET, serine/threonine-protein kinase B-Raf, and immunotherapy using anticancer drugs such as checkpoint inhibitors result in relatively high progression-free survival and total survival, compared to traditional anticancer drug treatments (for example, platinum-based chemotherapy). However, the prognosis of lung cancer patients remains poor [3].

The human lungs serve as a niche for a unique and dynamic bacterial community, characterized by the bi-directional movement of non-sterile air and mucus in the airways [4]. During respiratory diseases, significant differences in community composition between healthy and diseased lungs were observed [5]. Recent studies using next-generation sequencing have revealed that the lung microbiome in patients with lung cancer differs from that in healthy individuals [6–8]. Several studies have suggested a link between the lung microbiome and chronic lung diseases such as asthma, chronic obstructive pulmonary disease (COPD), cystic fibrosis, idiopathic pulmonary fibrosis, and respiratory infection [5]. For example, previous COPD studies on the microbiome showed that its severity increases as microbiome diversity decreases and that the microbiome affects the acute exacerbation of COPD [9]. Microbiota have been shown to modulate the efficacy and toxicity of cancer therapy, including chemotherapy and immunotherapy [10]. Preclinical data have suggested that modulation of the microbiota may present a novel strategy for improving the efficacy of immunotherapies for cancer, particularly, checkpoint blockade approaches targeting the cytotoxic T-lymphocyte-associated protein 4 and programmed cell death protein 1 (PD-1) pathways[11, 12].

Understanding the mechanisms through which microorganisms present in the respiratory tract may influence lung carcinoma development and treatment efficacy may be the key to predict the risk of cancer development and improve treatment efficacy and safety [13].

Microbial changes are thought to be associated with the accumulation of a PD-L1 dependent T regulatory cell population that promotes tolerance to environmental allergens [14]. Therefore, we hypothesized that the composition of the microbiome might differ based on PD-L1 expression levels. This study aimed to investigate the microbial differences in patients with lung cancer according to PD-L1 expression levels. In addition, we investigated whether there was a difference in the microbiome between responders and non-responders to immunotherapy.

## Methods

### Patient recruitment and sample collection

From June 1, 2018, to June 31, 2020, we prospectively recruited 84 patients who were pathologically diagnosed with non-small cell lung cancer (NSCLC) in two tertiary hospitals, Severance Hospital and Seoul National University Bundang Hospital, South Korea. Bronchoalveolar lavage (BAL) fluid samples were collected by a bronchoscopy specialist using a sterile bronchoscope. PD-L1 expression was measured from lung cancer biopsy via bronchoscopy, percutaneous CT-guided needle biopsy, and intraoperative thoracoscopy using the Tumor Proportion Score (TPS) by immunohistochemistry in tumor tissue. The cut-off value of PD-L1 expression was set to 10% according to a previous study showing that survival improvement with immunotherapy (nivolumab) was better than that with docetaxel for patients with PD-L1 expression ≥ 10% [15]. Detailed methods of sample collection are described in Additional file 2.

### DNA extraction, polymerase chain reaction (PCR) amplification, and sequencing

Total DNA was extracted using the FastDNA® SPIN Kit for Soil (MP Biomedicals, Santa Ana, CA, USA), according to the manufacturer’s instructions. PCR amplification was performed using fusion primers targeting V3 to V4 regions of the 16S rRNA gene of the extracted DNA. For bacterial amplification, fusion primers of 341F (5’-AATGATACGGCGACCACCGAGATCTACAC-XXXXXXXXTCGTCGGCAGCGTC-AGATGTGTATAAGAGACAG-CCTACGGGNGGCWGCAG-3’; the underlined sequence indicates the target region of the primer) and 805R (5’-CAAGCAGAAGACGGCATACGAGAT-XXXXXXXXGTCTCGTGGGCTCGG-AGATGTGTATAAGAGACAG-GACTACHVGGGTATCTAATCC-3’) were used. Amplification was performed under the following conditions: initial denaturation at 95 °C for 3 min, followed by 25 cycles of denaturation at 95 °C for 30 s, primer annealing at 55 °C for 30 s, and extension at 72 °C for 30 s, with a final elongation step at 72 °C for 5 min.

The PCR product was confirmed by performing 1% agarose gel electrophoresis and was visualized using a Gel Doc system (BioRad, Hercules, CA, USA). The amplified products were purified using the CleanPCR kit (CleanNA, Waddinxveen, Netherlands). Equal concentrations of purified products were pooled together, and short fragments (non-target products) were removed using CleanPCR. The quality and product size were assessed with Bioanalyzer 2100 (Agilent Technologies, Palo Alto, CA, USA) using a DNA 7500 chip. Mixed amplicons were pooled, and sequencing was performed at Chunlab, Inc. (Seoul, Korea) using an Illumina MiSeq Sequencing system (Illumina, San Diego, CA, USA) according to the manufacturer’s instructions. Further methods of taxonomic configuration are described in Additional file 2.

### Definition of chemotherapy response

The response to cancer treatment was divided into four categories according to the RECIST guideline [16]. Accordingly, patients who showed progressive disease (PD) after 3 months of cancer treatment were classified as non-responders, whereas patients who showed stable disease (SD), partial response (PR), and complete resolution (CR) were considered responders.

### Statistical analysis

Categorical variables are reported as numbers (percentages). Continuous variables with normal distribution are reported as means ± standard deviations, whereas variables with abnormal distribution are reported as medians with interquartile ranges (IQR, 25th to 75th percentiles). Categorical variables were compared using the chi-square test, and continuous variables were compared using either an independent t-test or Mann–Whitney U test according to the normality of distribution. A value of p < 0.05 indicated significance. All statistical analyses were performed using IBM SPSS Statistics version 25.0 (IBM, Armonk, NY, USA).

### Ethics approval and patient consent

The research protocol was approved by the Institutional Review Board of Severance Hospital, South Korea (IRB No. 4-0018-0313), and Seoul National University Bundang Hospital, South Korea (IRB No. B-1610/365-302). The study design was approved by the appropriate ethics review boards, and informed patient consent was obtained.

## Results

### Characteristics of the subjects

From June 1, 2018, to June 31, 2020, a total of 84 patients, who were pathologically diagnosed with non-small cell lung cancer (NSCLC) in two tertiary hospitals, Severance Hospital and Seoul National University Bundang Hospital, South Korea, were recruited in this prospective study.

The baseline characteristics of the two groups are shown in Table 1. The patients were stratified into two groups: 59 patients belonged to the low PD-L1 (< 10%) group, and 25 belonged to the high PD-L1 (≥ 10%) group. The mean age was 66.7 ± 11.2 years. Male was the dominant sex in both groups (61.0% vs. 64.0%); most of the patients had adenocarcinoma (76.3% in the low PD-L1 group vs. 76.0% in the high PD-L1 group), and the rest were diagnosed with squamous cell carcinoma. In the low PD-L1 group, the proportion of early-stage lung cancer was higher; contrastingly, patients with advanced-stage lung cancer were more abundant in the high PD-L1 group.

**Table 1:** Demographics and clinical characteristics of patients

Characteristics	PD-L1 < 10%	PD-L1 ≥ 10%	Total	P-value
No	59 (70.2)	25 (29.8)	84 (100.0)	
Age (year)	68.1 ± 10.7	63.1 ± 12.9	66.7 ± 11.2	0.080
Gender				0.797
Male, n (%)	36 (61.0)	16 (64.0)	52 (61.9)	
Female, n (%)	23 (39.0)	9 (36.0)	32 (38.1)	
Smoking history				0.574
Current or former, n (%)	32 (54.2)	16 (64.0)	48 (57.1)	
Never, n (%)	27 (45.8)	9 (36.0)	36 (42.9)	
Smoking amount (pack-years)	32.1 ± 15.6	30.2 ± 15.5	31.5 ± 15.4	0.687
Immunotherapy responder	6 (10.2)	2 (8.0)		
Immunotherapy non-responder	2 (3.4)	1 (4.0)		
Neutrophil–Lymphocyte ratio	2.93 (1.94, 6.13)	3.14 (1.76, 5.09)	3.05 (1.91, 5.85)	0.889
Stage				0.084
I/II/III/IV, n	24/5/14/16	3/4/9/9	27/9/23/25	
Pathologic diagnosis				0.979
Adenocarcinoma, n (%)	45 (76.3)	19 (76.0)	64 (76.2)	
Squamous cell carcinoma, n (%)	14 (23.7)	6 (24.0)	20 (23.8)	

PD-L1, programmed death-ligand 1

Of the 84 patients, the microbiome from 11 patients who underwent immunotherapy was analyzed (Table 2). Patients who showed SD, CR or PR after 3 months of immunotherapy were assigned to the responder group (eight patients), and patients showing PD were assigned to the non-responder group (three patients). The median age was 63.0 years.

**Table 2:** Demographics and clinical characteristics between the immunotherapy responder and non-responder groups

Characteristics	Responder	Non-responder	Total	P-value
No	8 (72.7)	3 (27.3)	11 (100.0)	
Age	63.0 (54.5, 73.8)	62.0 (50.0, 62.0)	63.0 (54.0, 74.0)	0.840
Gender				0.491
Male, n (%)	7 (87.5)	2 (66.7)	9 (81.8)	
Female, n (%)	1 (12.5)	1 (33.3)	2 (18.2)	
Smoking history				0.179
Current or former, n (%)	7 (87.5)	2 (66.7)	9 (81.8)	
Never, n (%)	1 (12.5)	1 (33.3)	2 (18.2)	
Pack-years	35.0 (30.0, 40.0)	41.5 (33.0, 41.5)	35.0 (30.0, 45.0)	0.526
Neutrophil–lymphocyte ratio	2.63 (2.13, 4.36)	1.98 (1.86, 1.98)	2.27 (1.98, 3.76)	0.133
PD-L1 ≥ 10%	2 (25.0)	1 (33.3)		
PD-L1 < 10%	6 (75.0)	2 (66.7)		
Stage				0.361
I/II/III/IV, n	0/0/4/4	0/0/1/2	0/0/5/6	
Pathologic diagnosis				0.661
Adenocarcinoma, n (%)	6 (75.0)	2 (66.7)	8 (72.7)	
Squamous cell carcinoma, n (%)	2 (25.0)	1 (33.3)	3 (27.3)	

### Characterization of the lung microbiome based on PD-L1 expression levels

**Fig. 1:**
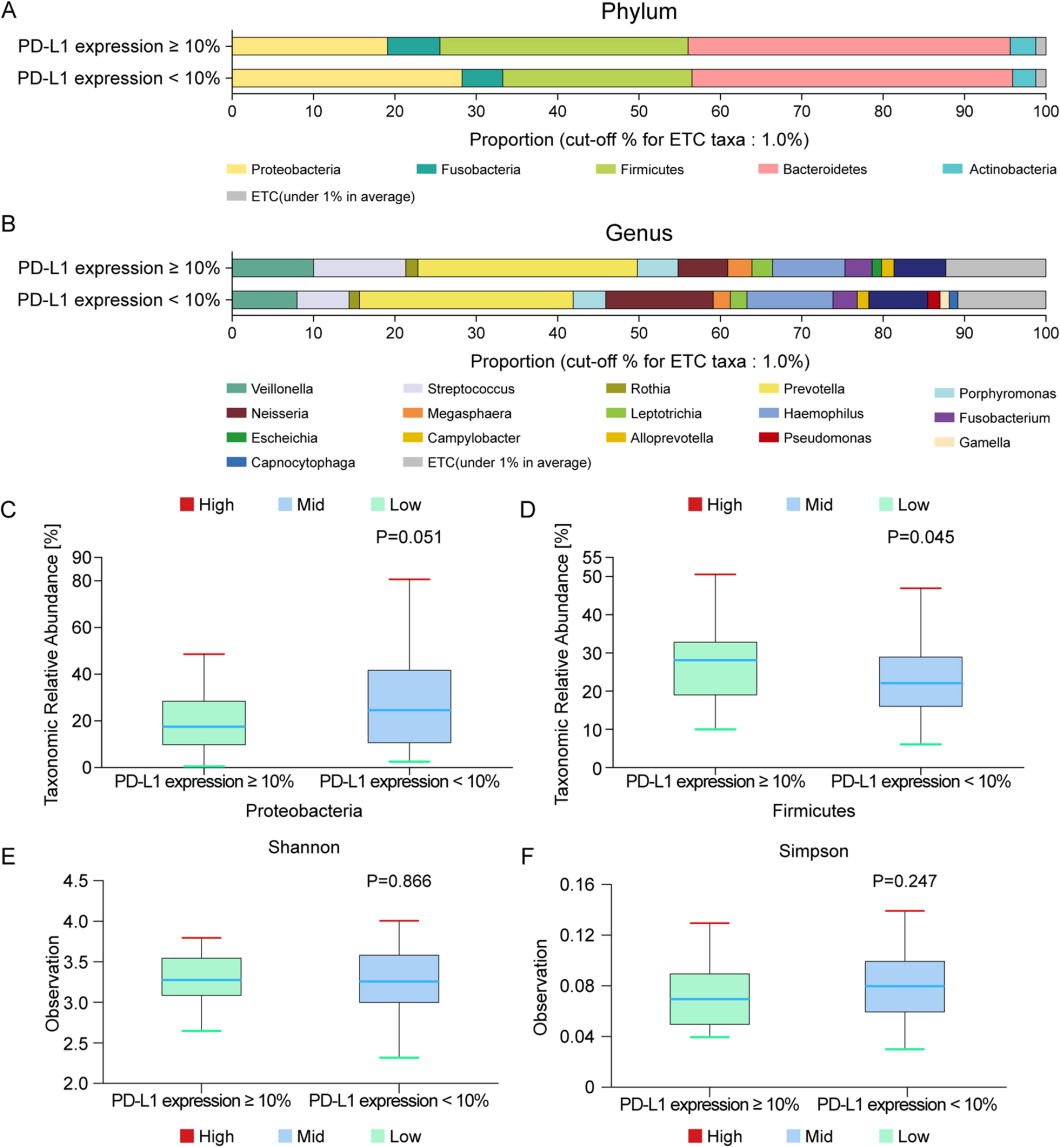
Taxonomic composition of the microbiome community between subgroups. **A** Dominant phyla based on PD-L1 expression levels (N = 84); high PD-L1 expression (N = 25); low PD-L1 expression (N = 59). **B** Dominant genera based on PD-L1 expression levels (N = 84); high PD-L1 expression (N = 25); low PD-L1 expression (N = 59). Differential abundances of the phyla **C** Proteobacteria and **D** Firmicutes based on PD-L1 expression levels. Comparison of the α diversity in bronchoalveolar lavage fluid microbiomes between the high- and low-PD-L1 expression groups. **E** Shannon index, **F** Simpson index. PD-L1, programmed death-ligand 1; upper box = 2nd quartile; mid line = median; lower box = 3rd quartile; whiskers = highest and lowest quartile

Figure 1a and b shows the differences in the lung microbiome according to PD-L1 expression levels. In the high-PD-L1 group, the dominant phyla were Bacteroidetes (39.4%), Firmicutes (30.5%), Proteobacteria (19.1%), Fusobacteria (6.4%), and Acinetobacter (3.2%). In the low-PD-L1 group, the phyla Bacteroidetes (39.4%), Proteobacteria (28.2%), Firmicutes (23.2%), Fusobacteria (5.1%), and Acinetobacter (2.8%) were dominant.

Proteobacteria and Firmicutes abundances differed between the low- and high-PD-L1 groups (Wilcoxon test, p = 0.051, Fig. 1c; p = 0.045, Fig. 1d). The Simpson index was evaluated to estimate α diversity in the lung microbiome that summarizes the structure of an ecological community with respect to its richness (number of taxonomic groups), evenness (distribution of abundances of the groups), or both [17]. The species richness differed between the two groups based on the Shannon index (p = 0.866, Fig. 1e) and Simpson index (p = 0.247, Fig. 1f), while the variability was not statistically significant. Principal coordinate analysis (PCoA) was performed to examine the similarity between bacterial communities of each group. As the operational taxonomic units (OTUs) of both low and high PD-L1 groups were clustered by each group, the bacterial community was similar, which is indicated by a dotted line. The Bray–Curtis distance was calculated to estimate the β diversity in the lung taxonomy community structure in patients with NSCLC, which metrics provide a measure of the degree to which samples differ from one another and can reveal the aspects of microbial ecology that are not apparent from the composition of individual samples[18]. However, there was no significant difference between the groups according to PD-L1 expression level (Additional file 1: Fig. S1).

**Fig. 2:**
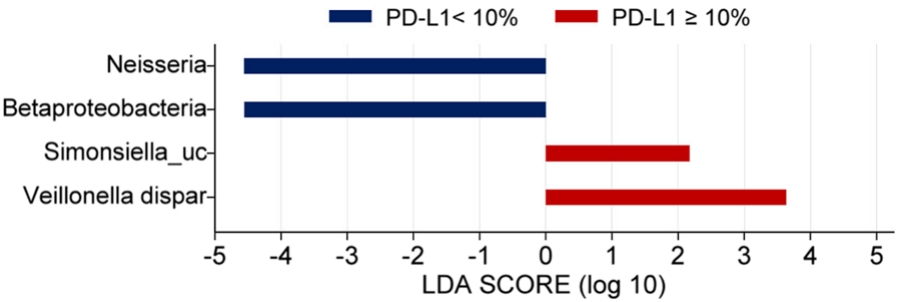
LEFse analysis of the collective dominant genera between the high- and low-PD-L1 expression level groups in patients with NSCLC. *LDA* linear discriminant analysis, *LEFse* LDA effect size, *PD-L1* programmed death-ligand 1. The blue bars indicate the taxa found in greater relative abundance in patients with low PD-L1 expression; the red bars indicate the taxa found in greater relative abundance in patients with high PD-L1 expression

LEFse analysis to further evaluate the differences in these dominant genera between patients with NSCLC in the low- and high-PD-L1 groups showed that the genus *Neisseria*, which belongs to the phylum Proteobacteria, was significantly more abundant in the low-PD-L1 group (Wilcoxon test, p = 0.037), and was also the genus with the greatest influence on the distinction between the two groups, with an LDA (linear discriminant analysis) score of 4.56 (Fig. 2). *Veillonella dispar,* belonging to the Firmicutes phylum, was dominant in the high-PD-L1 group (Wilcoxon test, p = 0.028, Fig. 2).

### Taxonomy composition in patients with lung cancer in the responder and non-responder groups

**Fig. 3:**
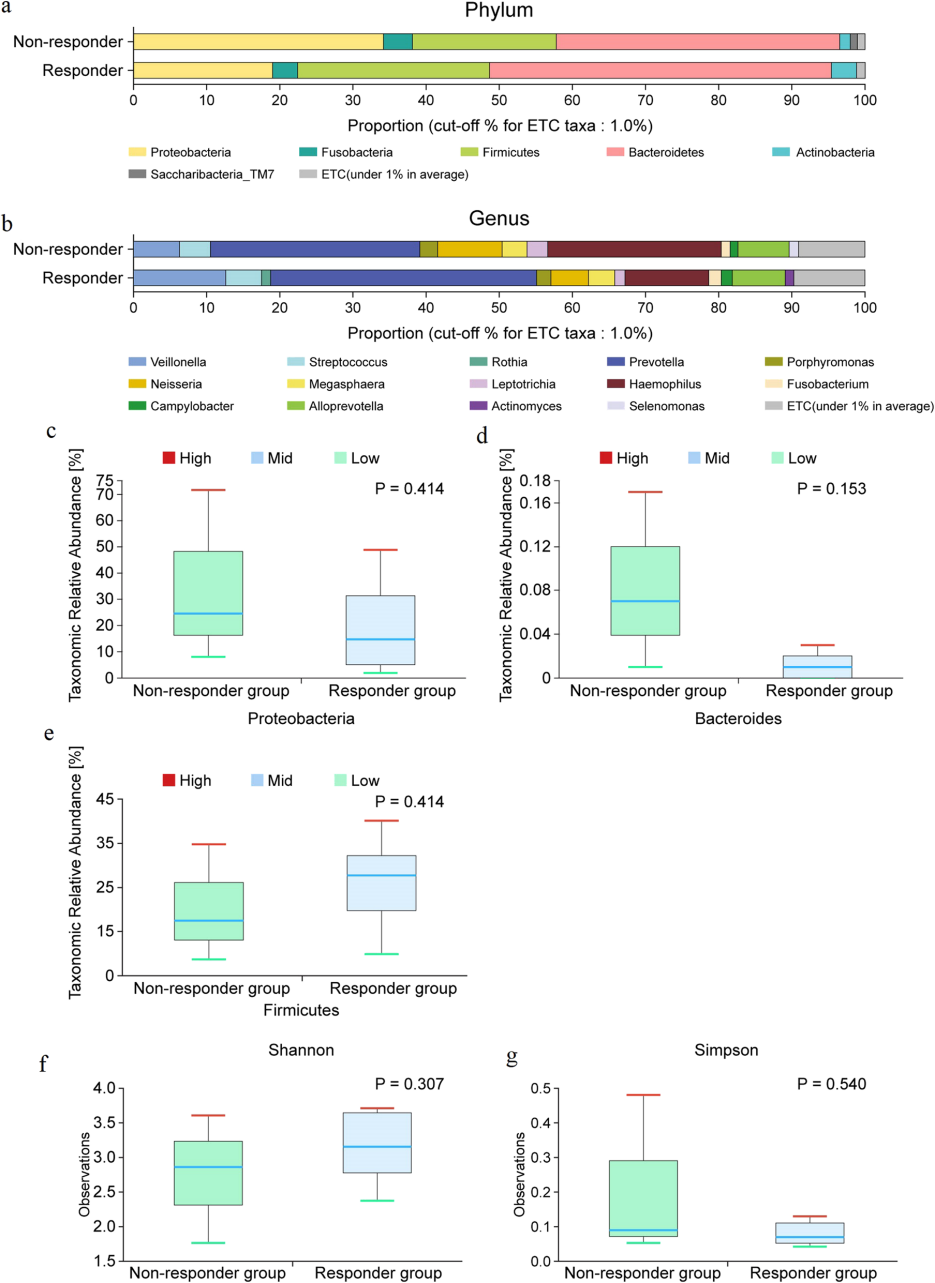
Taxonomic composition of the microbiome community between subgroups. **a** Dominant phyla based on response to immunotherapy (N = 11); Non-responder (N = 3), Responder (N = 8), **b** Dominant genera based on response to immunotherapy (N = 11); Non-responder (N = 3), Responder (N = 8). Differential abundances of phyla **c** Proteobacteria, **d** Bacteroides, and **e** Firmicutes between the immunotherapy responder and non-responder groups. Comparison of the α diversity in bronchoalveolar lavage fluid microbiomes between the responder and non-responder groups. **f** Shannon index, **g** Simpson index; upper box = 2nd quartile; mid line = median; lower box = 3rd quartile; whiskers = highest and lowest quartile

In the responder group, the dominant phyla were Bacteroidetes (46.8%), Firmicutes (26.2%), Proteobacteria (19.0%), Fusobacteria (3.5%), and Acinetobacter (3.4%). The dominant genera were *Prevotella* (36.4%), *Veillonella* (12.6%), *Haemophilus* (11.4%), *Alloprevotella* (7.3%), *Neisseria* (5.1%), *Streptococcus* (4.9%), and *Porphyromonas* (2.0%). Dominant phyla in the non-responder group included Bacteroidetes (36.8%), Proteobacteria (34.6%), Firmicutes (20.4%), and Fusobacteria (4.4%). Dominant genera included *Prevotella* (29.2%), *Haemophilus* (26.4%), *Neisseria* (6.7%), *Alloprevotella* (6.4%), *Veillonella* (6.2%), and *Megasphaera* (4.4%). The dominant phyla and genera in the immunotherapy responder and non-responder groups are shown in Fig. 3a and b.

Proteobacteria and Bacteroidetes populations were higher (Wilcoxon test, p = 0.414, Fig. 3c; p = 0.153, Fig. 3d), whereas that of the phylum Firmicutes was lower (Wilcoxon test, p = 0.414, Fig. 3e) in the non-responder group than in the responder group.

The Shannon and Simpson indices were evaluated to estimate the α diversity in the lung microbiome. The community in each microbiome was different; however, the difference was not significant (p = 0.307 for Shannon, Fig. 2f; p = 0.540 for Simpson index, Fig. 3g).

**Fig. 4:**
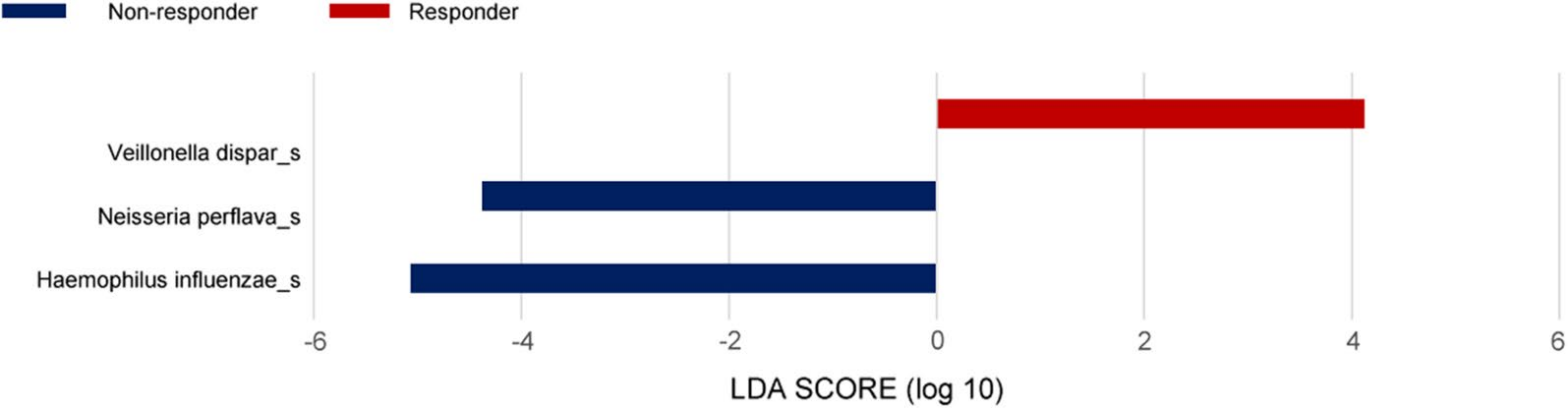
LEFse analysis of the dominant genera between the different immunotherapy response groups of patients with NSCLC. LDA, linear discriminant analysis; LEFse, LDA effect size. The blue bars indicate the taxa found in greater relative abundance in patients with low PD-L1 expression; the red bars indicate the taxa found in greater relative abundance in patients with high PD-L1 expression

We analyzed the differential taxonomy in BAL fluid samples between the patients with NSCLC in different response groups. LEFse analysis was performed to further evaluate the differences in the genera present in samples (Fig. 4). The population of *Haemophilus*, which belongs to the Proteobacteria phylum, was significantly higher in the non-responder group (Wilcoxon test, p = 0.041), whereas *Veillonella* was dominant in the responder group (Wilcoxon test, p = 0.041). It was also the genus that had the greatest effect on the difference between the responder and non-responder groups, with an LDA score of 5.1 for *Haemophilus influenzae* and 4.4 for *Neisseria perflava* in the non-responder group, and an LDA score of 4.1 for *V. dispar* (p = 0.041) in the responder group. The difference in the genera might have been related to the response to immunotherapy in patients with NSCLC.

## Discussion

In this study, we analyzed the bronchial microbiome in patients with NSCLC by performing 16S rRNA gene sequencing to analyze the relationship among microbiota composition, response to immunotherapy, and PD-L1 expression level. The ratio of Firmicutes to Bacteroidetes and the proportion of *Veillonella* were higher in the high-PD-L1 and immunotherapy responder groups than in the low-PD-L1 and non-responder groups.

In healthy individuals, the microbiota contributes to barrier function, communicates with the exterior, plays a role in immune homeostasis, and enhances anticancer immune surveillance via tumor antigenicity [19]. Additionally, the microbiota can trigger systemic innate immune responses via pattern recognition receptors, which further activate host responses against tumor cells [20].

Recent studies have shown that the gut microbiota affects the responses to immune checkpoint blockade therapy in patients with cancer [21–23]. The transplantation of fecal microbiota has been reported to augment and restore human immune responses, resulting in increased sensitivity to immunotherapy [24]. Recent studies have reported that organ-specific microbiomes, such as the lung microbiome, play an important role in lung cancer development [25].

Overall, our study did not show significant differences in the abundances of phyla in culture-independent DNA-based molecular assays; however, certain estimates of species richness showed significant differences in the PD-L1 expression groups.

There was a significant difference in culture-based genus types according to the differences in PD-L1 expression levels and immunotherapy responses. DNA sequencing analysis showed that the abundances of the genera *Neisseria*, *Veillonella*, and *Haemophilus* differed significantly between the two groups.

Regarding the LDA score indicating the effect size, the genus with the highest score in the low-PD-L1 group was *Neisseria* of the Proteobacteria phylum. In the non-responder group, the LDA score of *N. perflava* was significantly increased. It was previously reported that Proteobacteria abundance markedly increased in an anti-PD-L1 immunotherapy non-responder group of hepatocellular carcinoma [26]. However, the causal relationship of these correlations is not understood, and further research is needed.

*V. dispar* was dominant in the high-PD-L1 and immunotherapy responder groups. At present, studies show that the genus *Veillonella* is associated with the development of asthma [27, 28] and acute exacerbation of idiopathic pulmonary fibrosis [29]. Furthermore, Yan et al*.*[30] reported that *Veillonella* is abundant in patients with squamous cell carcinoma and adenocarcinoma of lung cancer, compared to healthy patients. They found that *Veillonella* may be used as a diagnostic marker to evaluate the development of both squamous cell carcinoma and adenocarcinoma [31]. Based on the findings of these studies, *Veillonella* present in samples collected from the airways may be associated with the development of multiple lung diseases. Our study also showed that the proportion of *Veillonella* was high in the responder group, but further studies are required as this study used a small sample size.

Tumor immunotherapy is performed not only to kill cancer cells but also to provide a long-term protective immunity mediated by memory CD8^+^ T cells [32, 33]. During cancer progression, the dysfunction in CD8^+^ T cell engagement and exhaustion owing to the tumor microenvironment results in an impairment in their function [34]. Identification of bacteria that directly or indirectly induce antitumor activities is crucial to developing microbiome-based combinatory treatments that can improve the overall response rate of anti-PD-1/PD-L1 treatment [35], which are targeted for T lymphocytes. We further demonstrated that the genus *Veillonella* was dominant in both high-PD-L1 and immunotherapy responder groups.

In this study, *Haemophilus,* which belongs to the phylum Proteobacteria, showed the largest LDA score of 5.1 in the non-responder group. It is known that most *Haemophilus* strains are opportunistic pathogens that coexist with the host without causing diseases and only cause diseases during viral infections or decreased immunity. Furthermore, a study showed that *Haemophilus* was more abundant in patients with lung cancer than in healthy individuals [36]. Our result showed that *Haemophilus* was abundant in the non-responder group; therefore, it can be inferred that members of this genus lower the effectiveness of anticancer drugs. Thus, *Haemophilus* may be considered as a target for disease-modulating drugs in lung cancer treatment.

The lung microbiome affects lung immunity and metabolism; several studies have shown that these microbial niches are associated with the pathogenesis of COPD, asthma, cystic fibrosis, and lung cancer [37–40]. Similarly, our study found that the microbiome was related to lung cancer treatment and showed the possibility of devising a method to increase the therapeutic effect using these microbiomes.

This study has a few limitations. First, the daily diet and antibiotic use in the patients, which could have affected the microbial composition, were not investigated in this study. However, bronchoscopy for obtaining the BAL fluid was performed at the time of first diagnosis; therefore, the effect of antibiotics was considered to be minimal. Second, the microbiome differed from one group to another; however, its role in cancer development is unclear. Third, we could not collect data from healthy controls and follow-up BALF to compare microbial differences after cancer treatment. Fourth, the sample size was small to obtain a strong correlation between the groups [responder (n = 8) vs. non-responder (n = 3)].

Despite these limitations, this study was conducted prospectively using 16S rRNA sequencing analysis, which is more sensitive and more informative than the conventional methods. More accurate and elucidating results can be obtained in large-scale studies.

## Conclusion

The abundances of *Neisseria* and *Veillonella* differed significantly in relation to PD-L1 expression levels and immunotherapy responses. *Haemophilus* was dominant in the immunotherapy non-responder group; however, the next-generation sequencing analysis did not show significant differences between the alpha and beta diversities in the lung microbiomes of patients in the immunotherapy responder group. Further larger cohort studies are needed to investigate the role of lung microbiome in lung cancer.

## Supplementary Information

**Additional file 1: Fig. S1.** PCoA plot based on Bray–Curtis distance of the BALF microbiome between the low-PD-L1 and high-PD-L1 expression groups.

**Additional file 2. **Additional methods.

## Abbreviations

PD-L1: Programmed death-ligand 1
NSCLC: Non-small cell lung cancer
LDA: Linear discriminant analysis
IQR: Interquartile ranges
PD: Progressive disease
SD: Stable disease
PR: Partial response
COPD: Chronic obstructive pulmonary disease
PD-1: Programmed death-protein 1
BAL: Bronchoalveolar lavage
LEFse: LDA effect size

## References

[1] Siegel RL, Miller KD, Jemal A (2020). Cancer statistics, 2020. CA Cancer J Clin.

[2] Barta JA, Powell CA, Wisnivesky JP (2019). Global epidemiology of lung cancer. Ann Glob Health.

[3] Yuan M, Huang LL, Chen JH, Wu J, Xu Q (2019). The emerging treatment landscape of targeted therapy in non-small-cell lung cancer. Signal Transduct Target Ther.

[4] Dickson RP, Huffnagle GB (2015). The lung microbiome: new principles for respiratory bacteriology in health and disease. PLoS Pathog.

[5] Dickson RP, Erb-Downward JR, Huffnagle GB (2013). The role of the bacterial microbiome in lung disease. Expert Rev Respir Med.

[6] Cameron SJS, Lewis KE, Huws SA, Hegarty MJ, Lewis PD, Pachebat JA, Mur LAJ (2017). A pilot study using metagenomic sequencing of the sputum microbiome suggests potential bacterial biomarkers for lung cancer. PLoS ONE.

[7] Tsay JCJ, Wu BG, Badri MH, Clemente JC, Shen N, Meyn P, Li Y, Yie TA, Lhakhang T, Olsen E (2018). Airway microbiota is associated with upregulation of the PI3K pathway in lung cancer. Am J Respir Crit Care Med.

[8] Apopa PL, Alley L, Penney RB, Arnaoutakis K, Steliga MA, Jeffus S, Bircan E, Gopalan B, Jin J, Patumcharoenpol P (2018). PARP1 is up-regulated in non-small cell lung cancer tissues in the presence of the cyanobacterial toxin microcystin. Front Microbiol.

[9] Wang Z, Bafadhel M, Haldar K, Spivak A, Mayhew D, Miller BE, Tal-Singer R, Johnston SL, Ramsheh MY, Barer MR (2016). Lung microbiome dynamics in COPD exacerbations. Eur Respir J.

[10] Panebianco C, Andriulli A, Pazienza V (2018). Pharmacomicrobiomics: exploiting the drug-microbiota interactions in anticancer therapies. Microbiome.

[11] Sivan A, Corrales L, Hubert N, Williams JB, Aquino-Michaels K, Earley ZM, Benyamin FW, Lei YM, Jabri B, Alegre M-L (2015). Commensal Bifidobacterium promotes antitumor immunity and facilitates anti-PD-L1 efficacy. Science.

[12] Vétizou M, Pitt JM, Daillère R, Lepage P, Waldschmitt N, Flament C, Rusakiewicz S, Routy B, Roberti MP, Duong CP (2015). Anticancer immunotherapy by CTLA-4 blockade relies on the gut microbiota. Science.

[13] Ramírez-Labrada AG, Isla D, Artal A, Arias M, Rezusta A, Pardo J, Gálvez EM (2020). The influence of lung microbiota on lung carcinogenesis, immunity, and immunotherapy. Trends Cancer.

[14] Gollwitzer ES, Saglani S, Trompette A, Yadava K, Sherburn R, McCoy KD, Nicod LP, Lloyd CM, Marsland BJ (2014). Lung microbiota promotes tolerance to allergens in neonates via PD-L1. Nat Med.

[15] Brahmer J, Reckamp KL, Baas P, Crinò L, Eberhardt WE, Poddubskaya E, Antonia S, Pluzanski A, Vokes EE, Holgado E (2015). Nivolumab versus Docetaxel in advanced squamous-cell non-small-cell lung cancer. N Engl J Med.

[16] Eisenhauer EA, Therasse P, Bogaerts J, Schwartz LH, Sargent D, Ford R, Dancey J, Arbuck S, Gwyther S, Mooney M (2009). New response evaluation criteria in solid tumours: revised RECIST guideline (version 1.1). Eur J Cancer.

[17] Willis AD (2019). Rarefaction, alpha diversity, and statistics. Front Microbiol.

[18] Goodrich JK, Di Rienzi SC, Poole AC, Koren O, Walters WA, Caporaso JG, Knight R, Ley RE (2014). Conducting a microbiome study. Cell.

[19] Zitvogel L, Ayyoub M, Routy B, Kroemer G (2016). Microbiome and anticancer immunosurveillance. Cell.

[20] Thaiss CA, Zmora N, Levy M, Elinav E (2016). The microbiome and innate immunity. Nature.

[21] Gopalakrishnan V, Spencer CN, Nezi L, Reuben A, Andrews MC, Karpinets TV, Prieto PA, Vicente D, Hoffman K, Wei SC (2018). Gut microbiome modulates response to anti-PD-1 immunotherapy in melanoma patients. Science.

[22] Matson V, Fessler J, Bao R, Chongsuwat T, Zha Y, Alegre ML, Luke JJ, Gajewski TF (2018). The commensal microbiome is associated with anti-PD-1 efficacy in metastatic melanoma patients. Science.

[23] Routy B, Le Chatelier E, Derosa L, Duong CPM, Alou MT, Daillère R, Fluckiger A, Messaoudene M, Rauber C, Roberti MP (2018). Gut microbiome influences efficacy of PD-1-based immunotherapy against epithelial tumors. Science.

[24] Baruch EN, Youngster I, Ben-Betzalel G, Ortenberg R, Lahat A, Katz L, Adler K, Dick-Necula D, Raskin S, Bloch N (2021). Fecal microbiota transplant promotes response in immunotherapy-refractory melanoma patients. Science.

[25] Goto T (2020). Airway microbiota as a modulator of lung cancer. Int J Mol Sci.

[26] Zheng Y, Wang T, Tu X, Huang Y, Zhang H, Tan D, Jiang W, Cai S, Zhao P, Song R (2019). Gut microbiome affects the response to anti-PD-1 immunotherapy in patients with hepatocellular carcinoma. J Immunother Cancer.

[27] Thorsen J, Rasmussen MA, Waage J, Mortensen M, Brejnrod A, Bønnelykke K, Chawes BL, Brix S, Sørensen SJ, Stokholm J, Bisgaard H (2019). Infant airway microbiota and topical immune perturbations in the origins of childhood asthma. Nat Commun.

[28] Espuela-Ortiz A, Lorenzo-Diaz F, Baez-Ortega A, Eng C, Hernandez-Pacheco N, Oh SS, Lenoir M, Burchard EG, Flores C, Pino-Yanes M (2019). Bacterial salivary microbiome associates with asthma among African American children and young adults. Pediatr Pulmonol.

[29] Molyneaux PL, Cox MJ, Wells AU, Kim HC, Ji W, Cookson WO, Moffatt MF, Kim DS, Maher TM (2017). Changes in the respiratory microbiome during acute exacerbations of idiopathic pulmonary fibrosis. Respir Res.

[30] Yan X, Yang M, Liu J, Gao R, Hu J, Li J, Zhang L, Shi Y, Guo H, Cheng J (2015). Discovery and validation of potential bacterial biomarkers for lung cancer. Am J Cancer Res.

[31] Rybojad P, Los R, Sawicki M, Tabarkiewicz J, Malm A (2011). Anaerobic bacteria colonizing the lower airways in lung cancer patients. Folia Histochem Cytobiol.

[32] Farhood B, Najafi M, Mortezaee K (2019). CD8(+) cytotoxic T lymphocytes in cancer immunotherapy: a review. J Cell Physiol.

[33] Ginefra P, Lorusso G, Vannini N (2020). Innate immune cells and their contribution to T-cell-based immunotherapy. Int J Mol Sci.

[34] Chihara N, Madi A, Kondo T, Zhang H, Acharya N, Singer M, Nyman J, Marjanovic ND, Kowalczyk MS, Wang C (2018). Induction and transcriptional regulation of the co-inhibitory gene module in T cells. Nature.

[35] Peng Z, Cheng S, Kou Y, Wang Z, Jin R, Hu H, Zhang X, Gong JF, Li J, Lu M (2020). The gut microbiome is associated with clinical response to anti-PD-1/PD-L1 immunotherapy in gastrointestinal cancer. Cancer Immunol Res.

[36] Druzhinin VG, Matskova LV, Demenkov PS, Baranova ED, Volobaev VP, Minina VI, Apalko SV, Churina MA, Romanyuk SA, Shcherbak SG (2020). Taxonomic diversity of sputum microbiome in lung cancer patients and its relationship with chromosomal aberrations in blood lymphocytes. Sci Rep.

[37] Dickson RP, Martinez FJ, Huffnagle GB (2014). The role of the microbiome in exacerbations of chronic lung diseases. Lancet.

[38] Huang D, Su X, Yuan M, Zhang S, He J, Deng Q, Qiu W, Dong H, Cai S (2019). The characterization of lung microbiome in lung cancer patients with different clinicopathology. Am J Cancer Res.

[39] Segal LN, Clemente JC, Tsay JC, Koralov SB, Keller BC, Wu BG, Li Y, Shen N, Ghedin E, Morris A (2016). Enrichment of the lung microbiome with oral taxa is associated with lung inflammation of a Th17 phenotype. Nat Microbiol.

[40] Sze MA, Dimitriu PA, Hayashi S, Elliott WM, McDonough JE, Gosselink JV, Cooper J, Sin DD, Mohn WW, Hogg JC (2012). The lung tissue microbiome in chronic obstructive pulmonary disease. Am J Respir Crit Care Med.

