# Supplementary figures and images for: Relationship of the lung microbiome with PD-L1 expression and immunotherapy response in lung cancer
Source: Respir Res. 2021 Dec 28;22:322. doi: 10.1186/s12931-021-01919-1 (PMC8715618; doi:10.1186/s12931-021-01919-1)

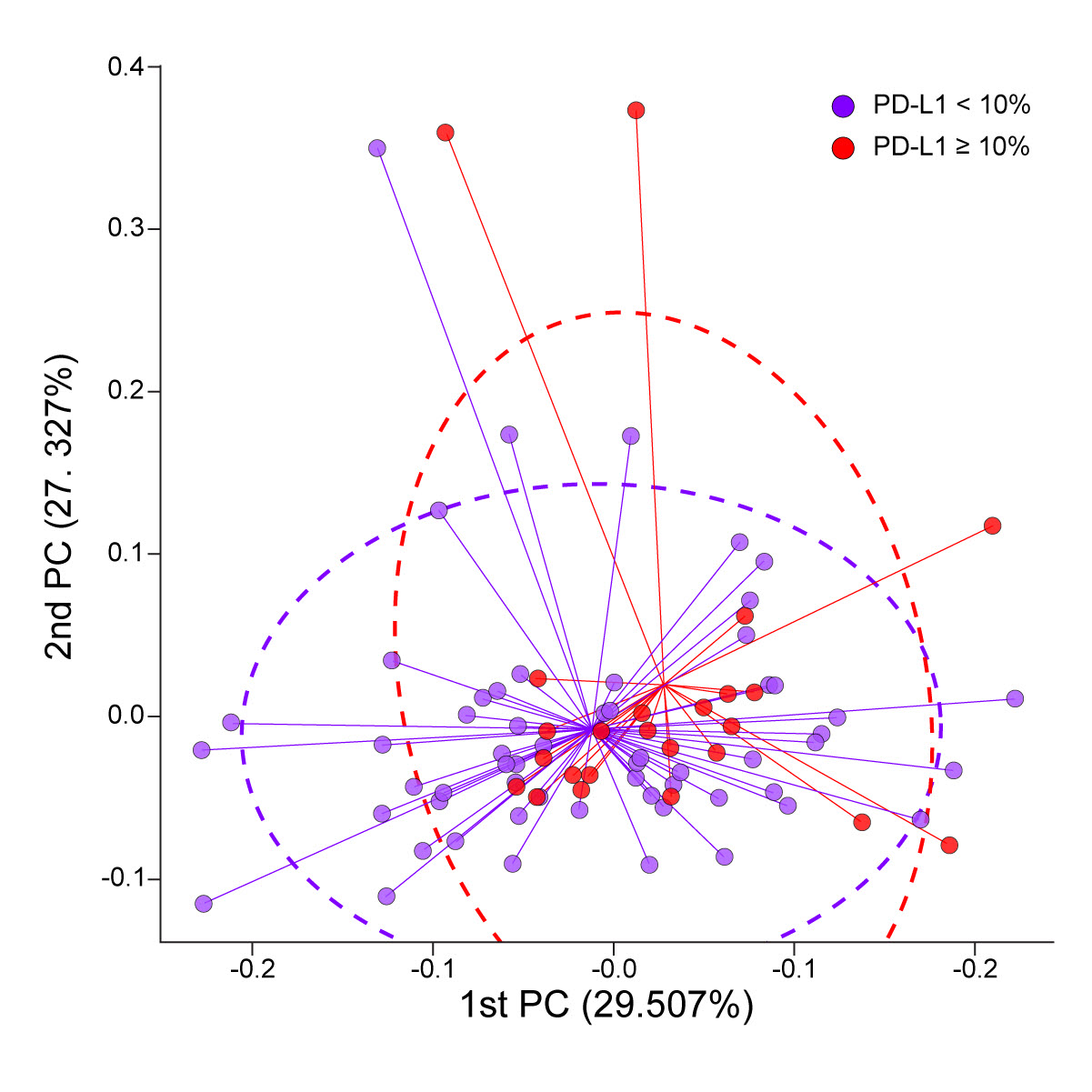

Supplement: Supplementary file 1 — Additional file 1: Fig. S1. PCoA plot based on Bray–Curtis distance of the BALF microbiome between the low-PD-L1 and high-PD-L1 expression groups. [file 12931_2021_1919_MOESM1_ESM.jpg]
