# Supplementary material for: Relationship of the lung microbiome with PD-L1 expression and immunotherapy response in lung cancer
Source: Respir Res. 2021 Dec 28;22:322. doi: 10.1186/s12931-021-01919-1 (PMC8715618; doi:10.1186/s12931-021-01919-1)
Supplement: Supplementary file 2 — Additional file 2. Additional methods. [file 12931_2021_1919_MOESM2_ESM.docx]

*Title: Relationship of the lung microbiome with PD-L1 expression and immunotherapy response in lung cancer*

Hye Jin Jang, MD^1^; Ji Yeon Choi, MD, PhD^1^, Kangjoon Kim, MD^1^, Seung Hyun Yong, MD^1^; Yeon Wook Kim, MD^2^; Song Yee Kim, MD^1^; Eun Young Kim, MD^1^; Ji Ye Jung, MD, PhD^1^; Young Ae Kang, MD, PhD^1^; Moo Suk Park, MD, PhD^1^; Young Sam Kim, MD, PhD^1^; Young-Jae Cho, MD, MPH, PhD^2,^*; Sang Hoon Lee, MD, PhD^1,^*

^1^Division of Pulmonology, Department of Internal Medicine, Institute of Chest Diseases, Severance Hospital, Yonsei University College of Medicine, Seoul, Republic of Korea

^2^Division of Pulmonary and Critical Care Medicine, Department of Internal Medicine, Seoul National University Bundang Hospital, Seongnam, Gyeonggi-do, Republic of Korea

**Supplemental methods**

*Sample collection*

Before the bronchoscopy, all patients rinsed their mouth twice with sterile saline. The patients then received a topical anesthesia (lidocaine) using a nebulizer. Subsequently, they were sedated with midazolam and fentanyl. The bronchoscope was wedged into the oral cavity of the patients, and then to the lungs. BAL fluid was obtained by following a standardized protocol. When the bronchoscope reached the “involved” airway containing the lung mass or the lung nodule, the bronchi were washed with 30–50 mL sterile saline (0.9%). A sample of approximately 15 mL BAL fluid was acquired from each patient for sequencing analysis. BAL fluid samples were immediately stored at -70 °C in a freezer, and DNA extraction was performed within 24 h.

Data Analysis Pipeline

For processing raw reads, a quality check (QC) was performed and low-quality reads (<Q25) were excluded using Trimmomatic ver. 0.32.[1] After the QC step, paired-end sequence data were merged together using the fastq_mergepairs command of VSEARCH version 2.13.4[2] with default parameters.

Primers were then trimmed using the alignment algorithm of Myers & Miller[3] at a similarity cut-off of 0.8. Non-specific amplicons that did not encode 16S rRNA were detected using the ‘nhmmer’ function of the HMMER software package ver. 3.2.1 with hidden Markov model profiles. Unique reads were extracted, and redundant reads were clustered with the unique reads using the derep_fulllength command of VSEARCH.[2] The EzBioCloud 16S rRNA database[4] was used for taxonomic assignment of the obtained 16S rRNA sequences using the ‘usearch_global ‘command of VSEARCH,[2] followed by a more precise pairwise alignment[3]. Chimeric reads were filtered from reads with <97% similarity via reference-based chimeric detection using the UCHIME algorithm[5] and the non-chimeric 16S rRNA database from EzBioCloud. After chimeric-read filtering, the reads that were not identified at the species level (with <97% similarity) using the EzBioCloud database, were compiled, and the ‘cluster_fast’ command[2] was used to perform de-novo clustering to generate additional operational taxonomic units (OTUs). Finally, OTUs with single reads (singletons) were omitted from further analysis. The secondary analysis, which included diversity calculation and biomarker discovery, was conducted using in-house programs of Chunlab, Inc (Seoul, South Korea). Shannon[6] and Simpson[6] alpha diversity indices were estimated. To visualize the sample differences, beta diversity distances were calculated using the method described by Bray–Curtis.[7] Taxonomic biomarkers and functional biomarkers were identified using statistical comparison algorithms (linear discriminant analysis [LDA] Effect Size [LEFse][8] and Kruskal–Wallis H Test[9]). To analyze the microbial community’s functional capabilities, functional profiling was conducted using PICRUSt (phylogenetic investigation of communities by reconstruction of unobserved states)[10] and MinPath (Minimal set of Pathways)[11]. All aforementioned analyses were performed using EzBioCloud 16S-based MTP, which is a Chunlab bioinformatic cloud platform.

**Figure legend**

**Supplemental Figure 1**. PCoA plot based on Bray–Curtis distance of the BALF microbiome between the low-PD-L1 and high-PD-L1 expression groups.

**References**

1. Bolger AM, Lohse M, Usadel B: **Trimmomatic: a flexible trimmer for Illumina sequence data.** *Bioinformatics* 2014, **30:**2114-2120.

2. Rognes T, Flouri T, Nichols B, Quince C, Mahé F: **VSEARCH: a versatile open source tool for metagenomics.** *PeerJ* 2016, **4:**e2584.

3. Myers EW, Miller W: **Optimal alignments in linear space.** *Comput Appl Biosci* 1988, **4:**11-17.

4. Yoon S-H, Ha S-M, Kwon S, Lim J, Kim Y, Seo H, Chun J: **Introducing EzBioCloud: a taxonomically united database of 16S rRNA gene sequences and whole-genome assemblies.** *International journal of systematic and evolutionary microbiology* 2017, **67:**1613.

5. Edgar RC, Haas BJ, Clemente JC, Quince C, Knight R: **UCHIME improves sensitivity and speed of chimera detection.** *Bioinformatics* 2011, **27:**2194-2200.

6. Magurran AE: *Measuring biological diversity.* John Wiley & Sons; 2013.

7. Beals EW: **Bray-Curtis ordination: an effective strategy for analysis of multivariate ecological data.** *Advances in ecological research* 1984, **14:**1-55.

8. Segata N, Izard J, Waldron L, Gevers D, Miropolsky L, Garrett WS, Huttenhower C: **Metagenomic biomarker discovery and explanation.** *Genome biology* 2011, **12:**1-18.

9. Kruskal WH, Wallis WA: **Use of ranks in one-criterion variance analysis.** *Journal of the American statistical Association* 1952, **47:**583-621.

10. Langille MGI, Zaneveld J, Caporaso JG, McDonald D, Knights D, Reyes JA, Clemente JC, Burkepile DE, Vega Thurber RL, Knight R, et al: **Predictive functional profiling of microbial communities using 16S rRNA marker gene sequences.** *Nature Biotechnology* 2013, **31:**814-821.

11. Ye Y, Doak TG: **A Parsimony Approach to Biological Pathway Reconstruction/Inference for Genomes and Metagenomes.** *PLOS Computational Biology* 2009, **5:**e1000465.
